# Human Antigen-Specific Regulatory T Cells Generated by T Cell Receptor Gene Transfer

**DOI:** 10.1371/journal.pone.0011726

**Published:** 2010-07-22

**Authors:** Todd M. Brusko, Richard C. Koya, Shirley Zhu, Michael R. Lee, Amy L. Putnam, Stephanie A. McClymont, Michael I. Nishimura, Shuhong Han, Lung-Ji Chang, Mark A. Atkinson, Antoni Ribas, Jeffrey A. Bluestone

**Affiliations:** 1 Diabetes Center, University of California San Francisco, San Francisco, California, United States of America; 2 Department of Surgery, University of California Los Angeles School of Medicine, Los Angeles, California, United States of America; 3 Department of Surgery, Medical University of South Carolina, Charleston, South Carolina, United States of America; 4 Department of Molecular Genetics and Microbiology, University of Florida College of Medicine, Gainesville, Florida, United States of America; 5 Department of Pathology and Laboratory Medicine, University of Florida College of Medicine, Gainesville, Florida, United States of America; University of Miami, United States of America

## Abstract

**Background:**

Therapies directed at augmenting regulatory T cell (Treg) activities *in vivo* as a systemic treatment for autoimmune disorders and transplantation may be associated with significant off-target effects, including a generalized immunosuppression that may compromise beneficial immune responses to infections and cancer cells. Adoptive cellular therapies using purified expanded Tregs represents an attractive alternative to systemic treatments, with results from animal studies noting increased therapeutic potency of antigen-specific Tregs over polyclonal populations. However, current methodologies are limited in terms of the capacity to isolate and expand a sufficient quantity of endogenous antigen-specific Tregs for therapeutic intervention. Moreover, FOXP3+ Tregs fall largely within the CD4+ T cell subset and are thus routinely MHC class II-specific, whereas class I-specific Tregs may function optimally *in vivo* by facilitating direct tissue recognition.

**Methodology/Principal Findings:**

To overcome these limitations, we have developed a novel means for generating large numbers of antigen-specific Tregs involving lentiviral T cell receptor (TCR) gene transfer into *in vitro* expanded polyclonal natural Treg populations. Tregs redirected with a high-avidity class I-specific TCR were capable of recognizing the melanoma antigen tyrosinase in the context of HLA-A*0201 and could be further enriched during the expansion process by antigen-specific reactivation with peptide loaded artificial antigen presenting cells. These *in vitro* expanded Tregs continued to express FOXP3 and functional TCRs, and maintained the capacity to suppress conventional T cell responses directed against tyrosinase, as well as bystander T cell responses. Using this methodology in a model tumor system, murine Tregs designed to express the tyrosinase TCR effectively blocked antigen-specific effector T cell (Teff) activity as determined by tumor cell growth and luciferase reporter-based imaging.

**Conclusions/Significance:**

These results support the feasibility of class I-restricted TCR transfer as a promising strategy to redirect the functional properties of Tregs and provide for a more efficacious adoptive cell therapy.

## Introduction

Natural regulatory T cells (nTregs), defined by expression of the transcription factors FOXP3 [1] and more recently Eos [2], play a critical role in maintaining immune tolerance [3]. Recent interest has been directed at this population as a means for providing cellular therapy in settings of both autoimmunity and transplantation [4]. In comparison, relatively non-specific therapies such as anti-CD3, anti-thymocyte globulin, cytokines, and adhesion molecule based agents are associated with significant non-specific immunological effects and side-effects. Despite these limitations, important mechanistic findings have emerged from these immune therapies. First, short term modulation of T cells can elicit long-term effects on immune tolerance [5]. Second, many of these agents thought to provide long-lived efficacy are considered to do so through promoting Treg subsets [6].

Treg transfers have been shown to effectively prevent or even reverse disease, which is especially interesting in settings of autoimmunity where such disorders are thought to result from the genetic absence or loss of Tregs from the periphery [7], [8]. A growing list of potential mechanisms of action for Tregs includes both cell-cell contact dependent and independent mechanisms (reviewed in [9]). From these numerous pathways, a common theme has emerged that Tregs function by multiple mechanisms at the site of antigen presentation; both in draining lymphoid tissue and at the site of inflammation, to create a regulatory milieu that promotes bystander suppression and infectious tolerance [10], [11]. In other words, activated Tregs are capable of suppressing cells recognizing common, as well as unrelated antigens in the local microenvironment and function to direct the development of activated conventional T cells (Tconv) toward a tolerant state. Discovering of the importance of Tregs in maintaining immune tolerance has opened a potentially new means of therapeutic intervention in immunology – namely, adoptive Treg therapy [12].

The concept of adoptive cell transfer initially emerged in the mid-1950s as a means to manipulate immune responses in the field of cancer therapy [13]. Cellular therapies have also been employed in patients with HIV using co-stimulated CD4^+^ T cells [14], as well as in the reconstitution of T cells following autologous and allogeneic stem cell transplantation [15]. While these treatments were designed to augment immune responses or stem cell recovery, a related approach involving transfer of T cells, specifically Tregs, has been employed to modify the immune response in autoimmune settings. The transfer of T cells from tolerized mice can prevent and/or reverse autoimmune disease [16]. The efficacy of transferred Tregs as a therapeutic modality has been demonstrated in animal models of systemic lupus erythematosus (SLE) [17], multiple sclerosis (MS) [18], inflammatory bowel disease (IBD) [19], oophoritis [20], and type 1 diabetes (T1D) [21], [22], [23], among others.

Although the therapeutic potential for Treg-based therapies is now well-established in animal models, to date adoptive Treg therapy has not been directly applied to suppress autoimmunity in humans. A number of challenges exist that need to be overcome in order to employ these cells in clinical settings, including the need for robust methods to isolate and expand this rare population. We recently described a clinically-relevant FACS-based isolation and *in vitro* expansion procedure for generating Tregs from patients with T1D using the markers CD4, CD25 (IL-2 receptor α-chain), and CD127 (IL-7 receptor α-chain) [24]. This approach was based on the observation that CD127 is inversely correlated with FOXP3 expression in humans [25], [26]. Utilizing this method, we demonstrated that isolation of the CD4^+^CD127^lo/−^CD25^+^T cell fraction yields an enriched population of Tregs that can be stably expanded *in vitro* without the need for additional selective agents [25].

As previously discussed, numerous examples exist that antigen-specific Tregs are more effective in several autoimmune syndromes when compared to polyclonal populations [21]. With this, a major challenge in antigen-specific Treg therapy in humans lies in the isolation of sufficient quantities of antigen-specific T cells - a challenge that is not easily overcome in humans where sampling is largely limited to peripheral blood. Limited but appreciable progress has recently been made to address this need including the development of reagents allowing for specific labeling of known antigen-specific T cells (e.g., peptide-MHC multimer staining of autoreactive cells); however, this approach is limited by several factors including the frequency of antigen-specific T cells in the periphery and the limited availability of appropriate peptide-MHC multimers. Adaptive Treg, such as those of the T_R_1- or T_H_3-type, can be generated from Tconv cell populations by culture under selective growth media conditions (e.g., rapamycin, retinoic acid, TGF-β or IL-10) [27]. Other efforts to overcome limited cell numbers have focused on delivering the transcription factor FOXP3 to Tconv cell populations [28]. However, both of these approaches to generate *de novo* Tregs are not without limitations. Specifically, adaptive Treg populations often lack the epigenetic profile of nTregs (i.e., they are often methylated at the *FOXP3*-Treg specific demethylation region (reviewed in [29]), bringing their stability *in vivo* into question. In the case of *FOXP3* gene transfer, expression of the transcription factor is required for Treg development, however transfer of *FOXP3* to Tconv cells does not recapitulate the full repertoire of transcripts and functional characteristics of nTregs [30]. Therefore, the approach we took was to initiate efforts with a fully mature polyclonal nTreg population isolated from peripheral blood and redirect cell specificity by viral TCR gene transfer. This approach has been employed previously as a cancer immunotherapy studies which showed that TCR α and β-chains could be introduced into CD4^+^ and CD8^+^ T cells in order to create a tumor-directed Teff cell population that upon adoptive transfer destroyed pre-existing tumor mass [31]. This approach has been shown to augment the therapeutic profile of mouse Tregs in an arthritis model [32], as well as redirect human Tregs using a chimeric immunoreceptor [33]. In this study, we demonstrate that the specificity of polyclonal human natural Tregs can be redirected by lentiviral TCR gene transfer. Moreover, we demonstrate in a proof-of-principal study that CD4^+^ Tregs can be engineered with class I-specific TCRs capable of suppressing immunity *in vivo*.

## Results

### Generation of a lentiviral expression vector expressing a tumor-specific TCR

**Figure 1:**
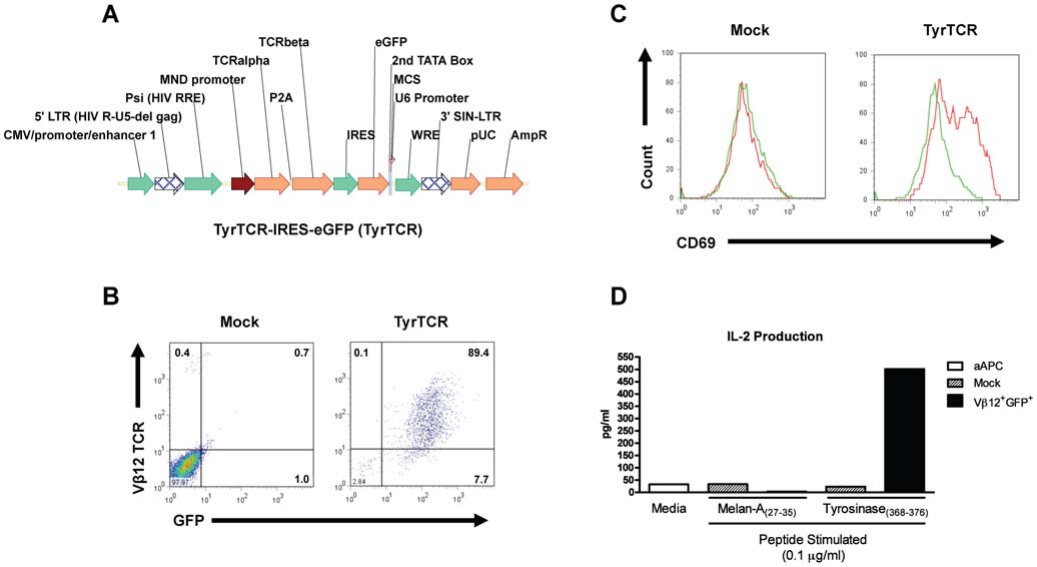
Lentiviral TCR expression construct and receptor validation in Jurkat T cells. (*A*) The tyrosinase TCR expression vector consists of a pSICO backbone, which has been modified to express the TCR α and β receptors derived from a CD4^+^ tumor infiltrating lymphocyte (clone TIL I383I). The resulting construct drives TCR-α and TCR-β proteins under the MND promoter from a single 2A peptide-linked tricistronic lentiviral vector followed by an eGFP reporter. (*B*) Human Jurkat T cells were transduced with the lentiviral construct by spin infection and sorted for the tyrosinase reactive TCR 72 h post-transduction. Sorted and Vβ12^+^GFP^+^ or mock transduced cells were then tested for their capacity to respond to their cognate peptide presented by artificial APCs (aAPCs). These K562-derived aAPCs present peptide in the context of human HLA-A*0201 along with the costimulatory/adaptor molecules CD80 and CD83. (*C*) Vβ12^+^GFP^+^ Jurkat T cells upregulate CD69 following 24 h exposure to [Asp^370^]-tyrosinase_(368–376)_ peptide (0.1 µg/ml) (*red histograms*) but not in response to the irrelevant HLA-A*0201 presented Melan-A/MART-1_(27–35)_ peptide (*green histograms*). (*D*) Culture supernatants from peptide stimulated Jurkat T cells were collected following 24 h of culture and analyzed for production of IL-2 by ELISA.

To express *de novo* TCRs in Tregs, we first generated an expression vector employing a previously characterized tumor-reactive TCR (TyrTCR) that recognizes the melanoma antigen tyrosinase in the context of the MHC class I molecule HLA-A*0201 [34]. The multicystronic expression vector allowed for equimolar TCR α and β chains by the inclusion of a T2A element [35] followed by an IRES element and eGFP reporter (**Figure 1A**). This receptor functions independently of the co-receptor; allowing for productive TCR signaling in CD4^+^ T cells [34], [36], [37]. Lentiviral transduction of Jurkat T cells was detected by flow cytometric analysis based on GFP reporter and tyrosinase-specific TCR Vβ12 expression (**Figure 1B**). Signaling by the introduced TCR was further validated by upregulation of CD69 and production of IL-2 following stimulation with artificial antigen presenting cells (aAPC, K562 cell line expressing HLA-A2, CD80, and CD83) and tyrosinase peptide (**Figure 1C,D**).

### Stability and functional capacity of TCR-redirected primary human CD4 T cells

**Figure 2:**
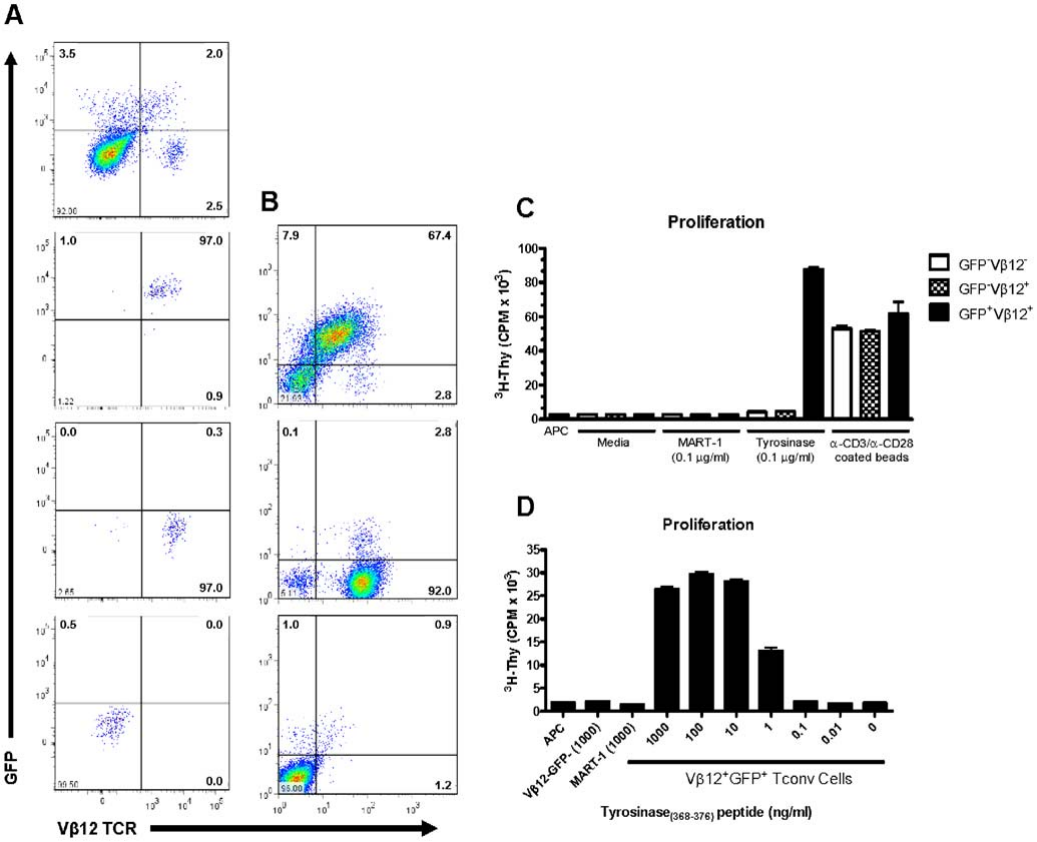
Isolation and characterization of lentiviral transduced primary human CD4^+^ T cells expressing the tyrosinase-reactive TCR. (*A*) Human CD4^+^ T cells were isolated by negative selection from fresh peripheral blood and activated for 48 h in the presence of anti-CD3 and anti-CD28 coated microbeads and human IL-2 (300 IU/ml). Cells were spin infected 48 h post-transduction and assessed for TCR Vβ12 and GFP expression by FACS 72 hr post-transduction (*A, upper-left hand plot*). Individual cell populations (column *A* plots) were FACS sorted based on expression TCR Vβ12 and GFP (Vβ12^+^GFP^+^, *upper-right quadrant*; Vβ12^−^GFP^+^, *lower-right*; Vβ12^−^GFP^−^, *lower-left*). Each sorted population was expanded in culture for 12 d and then assessed for stability of (*B*) TCR Vβ12 and GFP expression by FACS and (*C*) proliferation assessed by ^3^H-thymidine incorporation in response to peptide presented in the context of HLA-A*0201 expressing aAPCs. T cell populations (2×10^4^ cells/well) were stimulated by culturing 5×10^5^ irradiated (10,000 rads) aAPCs with soluble peptide as indicated (*C* and *D*). Proliferation is graphed as the mean±SD from triplicate wells. Data are representative of two independent experiments.

Given our ability to express a functional TCR in Jurkat T cells, we next sought to validate expression in primary human CD4^+^ T cells. Freshly isolated human CD4^+^ T cells were activated for 48 hr with anti-CD3 and anti-CD28-coated beads prior to transduction with lentivirus expressing the TyrTCR. GFP and TCR Vβ12 co-expressing cells were isolated by fluorescence-based cell sorting (**Figure 2A**). Expression of surface TCR was stable following *in vitro* expansion (**Figure 2B**) and the TyrTCR-transduced cells responded specifically to tyrosinase peptide in a dose-responsive fashion. No proliferation was observed from the native GFP^−^Vβ12^+^ sorted population stimulated with tyrosinase peptide or from the GFP^+^Vβ12^+^ T cell population incubated with an irrelevant HLA-A*201-restricted control peptide (MART-1) (**Figure 2C,D**). Cytokine production (IFN-γ, IL-10) by TyrTCR-transduced cells was also detected following co-culture with a melanoma tumor cell line expressing tyrosinase naturally processed and presented in the context of HLA-A*0201 (data not shown). The successful *de novo* expression of a functional TCR in primary human CD4^+^ T cells facilitated the subsequent targeting of this receptor to Tregs.

### Generation of engineered human Treg and Tconv cells

We recently described a clinically-relevant FACS-based isolation and *in vitro* expansion procedure for generating Tregs enriched in FOXP3 expression in humans [25]. Utilizing this method, we demonstrated that isolation of the CD4^+^CD127^lo/−^CD25^+^ T cells yielded a highly enriched population of Tregs that are greater than 90–95% FOXP3^+^ [24]. Subsequent studies suggested that the inclusion of CD45RA as a positive selection marker resulted in greater enrichment of FOXP3^+^ T cells and maximum stability following *in vitro* expansion [38], [39] (**Figure S1**). The purity and stability of this sorted population was further validated by assessing FOXP3 protein and FOXP3 promoter methylation status following *in vitro* expansion. Prior reports have suggested that only *bona-fide* nTregs remain demethylated at the *FOXP3* gene Treg-specific demethylation region (TSDR) [40]. CD45RA^+^ Tregs were determined to be FOXP3^+^ by FACS (mean+SD, 92.3+9.6%, N = 8) and demethylated at the TSDR (91.0+15.3%) following a 14 day expansion period, demonstrating the specific expansion of the nTreg subset (**Figure S1G**).

Next, the polyclonal population of purified nTregs was infected with lentivirus expressing a GFP reporter following activation as described in Materials and Methods. High levels of GFP expression were detected following *in vitro* expansion with no significant reduction in expansion capacity or suppressive function (**Figure S2**). Given the high efficiency of transgene expression in Tregs, we subsequently transduced a population of purified naïve Tregs and Tconv cells with a lentiviral TyrTCR construct. Freshly sorted cells were activated for 48 hr *in vitro* prior to a single infection with lentivirus encoding the TyrTCR. Surface TCR and tetramer staining, along with GFP reporter expression were analyzed by flow cytometry at day nine of the *in vitro* expansion period. Initial transduction of Tregs resulted in a degree of heterogeneity in the level of TCR and GFP reporter expression. Flow cytometric analysis indicated 20.7±16.6% of cells expressed eGFP, whereas 13.2±14.5% of cells were GFP^+^Vβ12^+^ following nine days of *in vitro* expansion (*N* = 10). Surface expression of TCR Vβ12 might not indicate functional receptor expression, given the possibility for heterologous pairing with endogenous TCR α and β chains [41]. To further validate proper surface expression, HLA-A*201-tyrosinase tetramer staining of transduced Tregs revealed 33.5% of cells were GFP^+^TyrTCR tetramer^+^, whereas 16.8% of Tconv cells were GFP^+^TyrTCR tetramer^+^ (**Figure S3B**).

**Figure 3:**
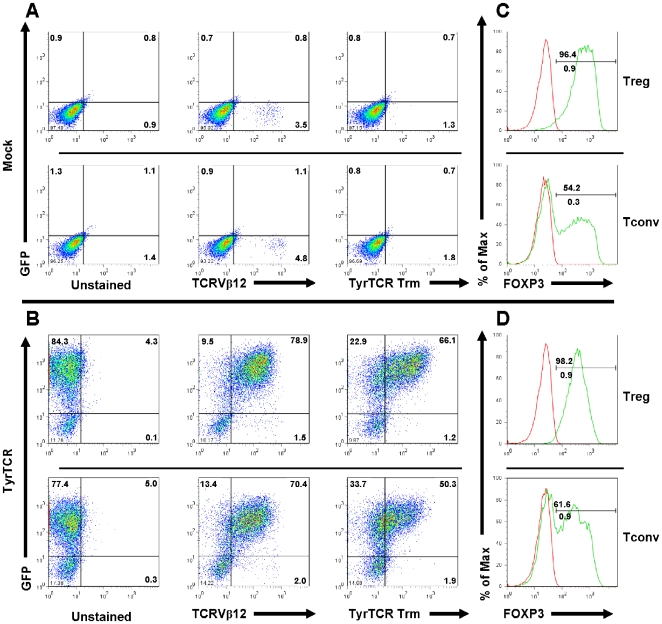
Expression of tyrosinase TCR constructs by *in vitro* expanded human Treg and Tconv cells. Plots indicate expression of surface TCR, GFP, and FOXP3 following 14 day *in vitro* expansion period for mock or TyrTCR transduced Treg and Tconv cells. Fresh human PBMCs were sorted by FACS to yield CD4^+^CD127^−/lo^CD25^+^CD45RA^+^ Tregs and CD4^+^CD127^+^CD25^−^CD45RA^+^ Tconv cell populations and stimulated with anti-CD3 and anti-CD28 microbeads for 48 hours prior to lentiviral spinoculation. Following eight days of *in vitro* expansion, mock transduced cells were restimulated with microbeads and TyrTCR transduced T cells were enriched by restimulation with aAPCs and soluble tyrosinase peptide (0.1 µg/ml). Plots indicate expression of eGFP (*y-axis*) and TCR Vβ12 or HLA-A2 tyrosinase_(368–376)_ tetramer staining (*x-axis*) with (*A*, *upper plots*) representing mock transduced cells and (*B*, *lower plots*) showing TyrTCR transduced populations. Histograms overlays indicate isotype control staining (*red histograms*) or FOXP3 staining (*green histograms*) of mock or TyrTCR transduced populations. Data shown represent one subject of ten independent experiments following lentiviral TyrTCR transduction of Tregs.

We observed that increasing lentiviral titer led to only modest increases in the number of cells expressing functional receptors (data not shown). Therefore, we sought to enrich antigen-specific T cells while limiting non-specific growth, as previously described [42]. At day nine, cells expressing the TyrTCR were restimulated with aAPCs and tyrosinase peptide. This antigen-specific activation led to enrichment of the transduced cells with 66.1% of Treg and 50.3% of Tconv cells expressing both GFP and the TyrTCR (**Figure 3A,B**). Mock transduced cells were restimulated by anti-CD3 and anti-CD28 coated beads. The expansion capacity of TyrTCR Tregs expanded on aAPC and peptide was dependent upon the initial transduction efficiency (*N* = 3; median 480-fold, range 240 to 1088-fold). Of note, we were able to obtain >5200-fold expansion of TyrTCR^+^ Tregs yielding 1.18×10^9^ cells (89.3% eGFP^+^, 91.7% FOXP3^+^), from an initial blood volume of 80 ml by extending the culture to day 20 (data not shown).

Following expansion, 96.4% of mock-transduced Tregs stained positive for FOXP3 protein by FACS with 100% of cells remaining demethylated at the *FOXP3*-TSDR for the sample shown **(** **Figure 3C**, *upper panel*). By comparison, 98.2% of TyrTCR-transduced Tregs stained positive for FOXP3 protein by FACS with 99.1% of cells remaining demethylated at the *FOXP3*-TSDR (**Figure 3D**, *upper panel*). As a population, Tregs expressing the TyrTCR expressed comparable levels of FOXP3 (95.6±2.0) in comparison to mock Tregs (93.6±2.3; *N* = 8, *P* = not significant). It is interesting to note that Tconv cells expanded poorly under our Treg growth conditions and tended to upregulate FOXP3 protein as determined by FACS (33.8±17.9 vs 35.9±22.8 for mock and TyrTCR, respectively, *N* = 3). Despite this induction of FOXP3 protein, epigenetic analysis of the *FOXP3*-TSDR indicated the absence of *bona-fide* Tregs (0.02% and 2.81% demethylated for mock and TyrTCR Tconv cells, respectively, **Figure 3C,D**). This observation highlights the need for monitoring of the methylation status of the *FOXP3*-TSDR as a stringent quantitative measure of cell purity post-expansion [43].

### Functional capacity of engineered Tregs

**Figure 4:**
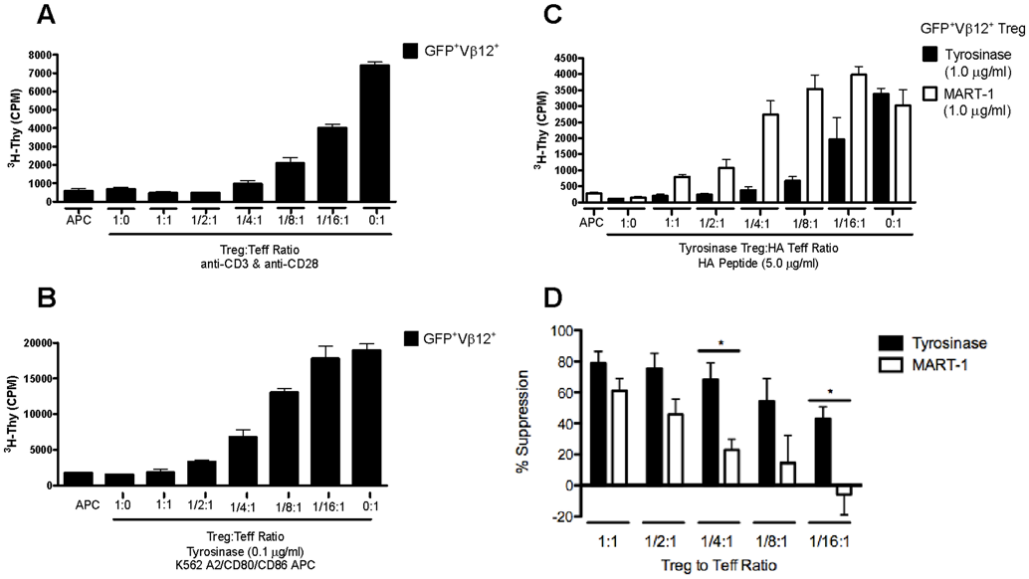
*In vitro* suppressive capacity of lentiviral transduced Tregs expressing the tyrosinase TCR. TyrTCR transduced Treg and Tconv cells were sorted to enrich the GFP^+^Vβ12^+^ cell population (as indicated in Fig. 2A). The sorted Tregs were then tested for their capacity to suppress proliferation of autologous expanded Tconv cells during *in vitro* suppression assays following both polyclonal and antigen-specific T cell activation. (*A*) FACS sorted GFP^+^Vβ12^+^ Treg cells suppress proliferation of anti-CD3 and anti-CD28 stimulated GFP^+^Vβ12^+^ Tconv cells in a dose-responsive manner. (*B*) *In vitro* expanded Tregs suppress TyrTCR GFP^+^Vβ12^+^ Tconv cells stimulated by aAPCs and tyrosinase peptide. (*C*) A representative suppression assay demonstrating GFP^+^Vβ12^+^ Tregs are more potent at suppressing autologous hemagglutinin (HA)-reactive Teff cells when stimulated by their cognate antigen compared to an irrelevant HLA-A2 restricted peptide. HA-reactive Teff cells were stimulated by HLA-DR*0401 expressing APCs and HA peptide with Tregs stimulated with either tyrosinase peptide (*C, closed bars*) or an irrelevant HLA-A*0201 restricted peptide melan-A (MART-1) (*C, open bars*). (*D*) Percent suppression of HA-reactive Teff cells stimulated in the presence of Tyr-Tregs activated by tyrosinase peptide (*black bars*) or MART-1 (*open bars*) (*N* = 3 independent experiments, *P<0.05). Shown are mean±SEM.

The capacity of expanded TCR-transduced Tregs to suppress proliferation of freshly isolated conventional CD4^+^ T cells was examined following stimulation with anti-CD3 and anti-CD28. GFP^+^TyrTCR^+^ Tregs were FACS sorted following 14 days of *in vitro* expansion and mixed at indicated ratios of Tregs to Tconv cells. TyrTCR Tregs suppressed polyclonal T cell proliferation potently and in a dose-responsive fashion (**Figure 4A**). We next tested the capacity of TyrTCR Tregs to suppress antigen-specific CD4^+^CD127^+^CD25^−^ Tconv cell responses. Following expansion, Treg and Tconv GFP^+^TyrTCR^+^ cells were sorted from expanded cultures and restimulated with tyrosinase peptide in the presence of aAPCs (**Figure 4B**). TyrTCR-expressing Tregs were capable of suppressing Teff cell responses in an antigen-specific fashion.

The therapeutic efficacy of Tregs depends on their capacity to suppress an array of T cell responses in a bystander fashion, as well as antigen-specific fashion. In order to address this issue, we tested the capacity of TyrTCR-transduced Tregs to suppress proliferation of hemagglutinin (HA) peptide-reactive Teff cells. To generate human HA-reactive Teff cells, we labeled PBMCs from a HLA-DR*0401 individual with CFSE and stimulated with HA peptide_(306–318)_ to induce expansion of endogenous HA-specific T cells. Following 14 day expansion, CFSE-diluted cells (>13% HLA-DR*0401 HA-tetramer^+^; data not shown) were used as responding Teff cells in subsequent assays. For suppression assays, TyrTCR Tregs were stimulated in the context of HLA-A*0201, while the responding Teff cells were stimulated in the context of HLA class II DR*0401 APCs incubated with HA peptide_(306–318)_. To test the specific activation requirements of Tregs in mediating bystander suppression, TyrTCR-transduced Tregs were stimulated with tyrosinase peptide or the irrelevant HLA-A*0201 restricted MART-1 peptide. This analysis indicated that TyrTCR-transduced Tregs were indeed capable of suppressing HA-reactive Teff cell proliferation in a bystander fashion and demonstrated that TyrTCR cells maintained optimal potency when activated specifically through the introduced TCR (**Figure 4C**). This was particularly apparent at lower and more physiological ratios of Tregs to Teff cells (i.e. ^1^/_4_∶1, ^1^/_8_∶1, and ^1^/_16_∶1) (**Figure 4D**). Despite this increased potency, we observed some antigen non-specific suppression by TyrTCR Tregs *in vitro* (particularly at higher Treg to Teff cell ratios, i.e. 1∶1), suggesting that Tregs may not be fully rested following the activation received during the *in vitro* expansion protocol. We also analyzed the ability of TyrTCR Tregs to influence cytokine production (e.g. IFN-γ, IL-2, and IL-10) when co-incubated with a tumor infiltrating cell line (TIL1235) and peptide pulsed APCs. Consistent with our prior investigations [24], [44], TyrTCR transduced Tregs suppressed effector T cell cytokine production in a dose-responsive fashion for IFN-γ and IL-2. Interestingly, the reciprocal cytokine production profile was seen for IL-10, wherein co-cultures with TyrTCR Tregs, but not mock Tregs produced the highest levels of this immunoregulatory cytokine (**Figure S4**). While *in vitro* suppressive activity is an important indication of Treg function, it does not reflect the full activity of Tregs *in vivo*. Therefore, we sought to test whether TCR-transduced Tregs were capable of suppressing T cell-mediated tissue damage *in vivo*.

### *In vivo* activity of TyrTCR expressing Tregs

**Figure 5:**
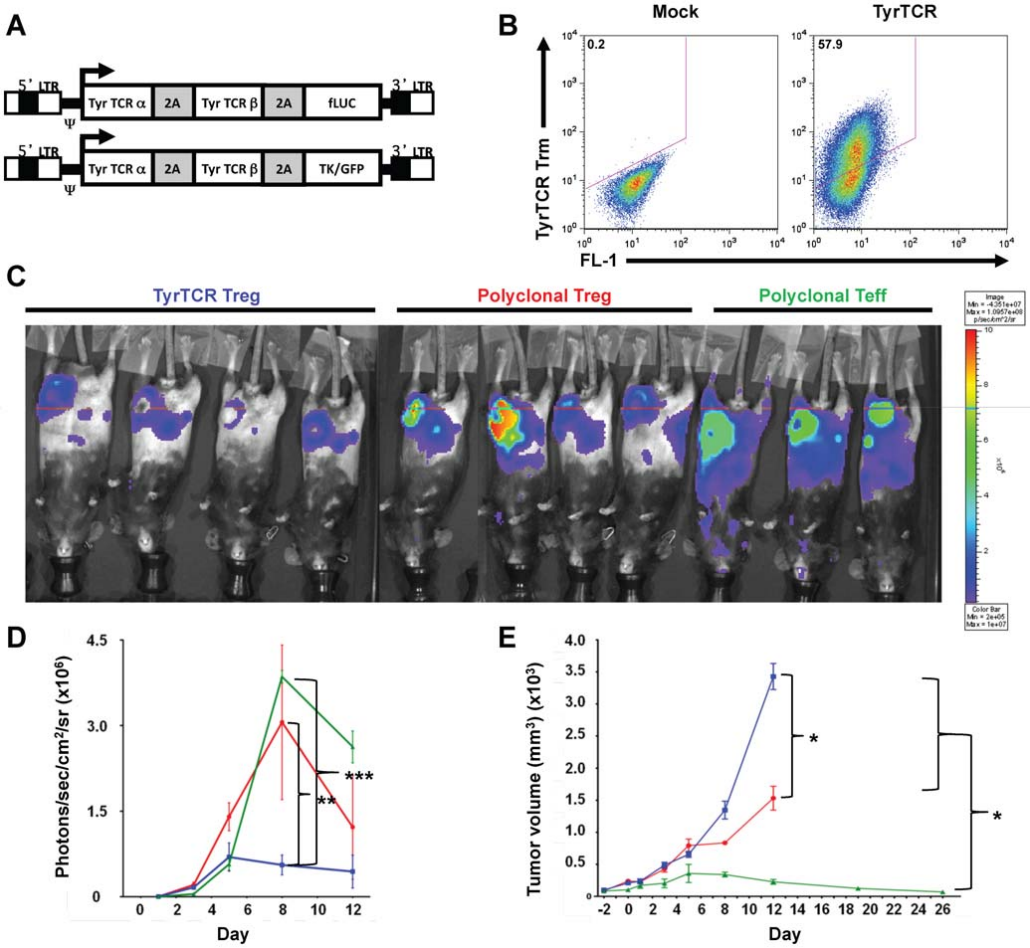
Tyrosinase TCR transduced Tregs block antigen-specific effector T cell activity *in vivo*. Eight-weeks old HLA-A2/K^b^ transgenic mice were injected subcutaneously (s.c.) with EL4-A2/K^b^ tyrosinase protein expressing tumors (5 mm in diameter) in the inguinal fat pad. Murine T cells were transduced with MSCV-based retroviral vectors encoding the Tyrosinase TCR with (*A*) Firefly Luciferase (fLuc) reporter for Teff cells or HSVtk/GFP reporter for Tregs. Mice were randomized into three groups receiving *in vitro* expanded Tregs transduced with TyrTCR/TK/GFP vector (TyrTCR Tregs), polyclonal mock Tregs (Untrd, Treg), or mock T effector cells (Untrd, Teff). (*B*) Mock or HLA-A*0201_(368–376)_ tetramer staining of TyrTCR transduced Tregs prior to injection. Following 24 hours, all mice received Teff cells transduced with Tyr-TCR/fLuciferase reporter. (*C*) Luciferase-based *in vivo* bioluminescence imaging (BLI) was performed for imaging of fLuc-transduced Teff cells. Fluorescence images of treatment groups are shown at day 8. (*D*) The mean number of photons per square centimeter per second per steradian in the region of interest (ROI) was determined on tumor-implanted sites for mice receiving TyrTCR Tregs (*blue square*), Untransduced Tregs (*red circle*), and Untrd Teff (*green triangle*). (*E*) Tumor site volume (mm^3^) assessed by caliper measurement for adoptive cell transfer treatment groups. Data shown is representative of two independent experiments with *P<0.05, **P<0.01, and ***P<0.0001 assessed by Two-way ANOVA with Bonferroni correction.

We employed a tumor model system to assess the *in vivo* function of TyrTCR-redirected Tregs. EL-4-HLA-A2/K^b^ tumors expressing tyrosinase were transferred into HLA-A2/K^b^ transgenic mice. Mice were then separated into groups receiving *in vitro* expanded murine TyrTCR Tregs, mock vector-transduced Tregs, or control vector-transduced Teff cells via intravenous injection. All mice received antigen-specific Teff cells expressing the TyrTCR. These TyrTCR Teff cells could be tracked *in vivo* by a firefly-Luciferase (fLuc) reporter element encoded within the expression construct (**Figure 5A**). The murine TyrTCR expression construct was modified for optimized surface expression and improved tetramer reactivity (R. Koya, unpublished observations). Specifically, the TyrTCR construct was modified to express murine TCR constant regions and human TCR variable regions [34]. Additional modifications consisted of introducing cysteine residues to the TCR α and β chains to induce disulfide linkages, 2A peptide linker sequences, and leucine zipper motifs between TCR chains as described in Materials and Methods. The modified TyrTCR could be expressed on murine CD4^+^ T cells with expression by expanded Tregs verified by HLA-A*201-Tyrosinase_(368–376)_ tetramer staining following *in vitro* expansion with anti-CD3 and anti-CD28-coated microbeads and IL-2 (**Figure 5B**).

We monitored the expansion and distribution of TyrTCR-expressing Teff cells using an *in vivo* luciferase reporter in treatment groups receiving TyrTregs, mock-vector transduced Tregs, or mock-vector transduced Teff cells. Imaging during the experimental time-course revealed broad TyrTCR Teff cell distribution and tumor infiltration in mice receiving either polyclonal Tregs or Teff cells (**Figure 5C**). In contrast, TyrTCR Teff cell expansion was restricted in mice receiving antigen-specific TyrTCR Tregs. This was most apparent at day eight post transfer with 5.6×10^5^, 3.1×10^6^, and 3.9×10^6^photons/sec/cm^2^/sr detected for TyrTCR Treg, polyclonal Treg, and Teff cells, respectively (**Figure 5D**). No significant difference in luciferase reporter was detected between Teff and polyclonal Treg treated mice.

TyrTCR-redirected Teff cells are highly effective at facilitating tumor regression upon transfer. We tested the capacity of antigen-specific Tregs to block tumor immunity in mice receiving TyrTCR Tregs, polyclonal Tregs, or control Teff cells. Mice receiving TyrTCR-redirected Tregs effectively blocked TyrTCR Teff cell-mediated tumor rejection as determined by tumor size (**Figure 5E**). In contrast, a partial suppressive effect was observed in mice treated with polyclonal Tregs, with near complete tumor rejection observed in mice receiving Teff cells alone. These data argue that TCR-redirected Tregs are indeed capable of suppressing Teff cell responses *in vivo*, and further demonstrate that antigen-specific Tregs are more potent than polyclonal Tregs in limiting Teff-cell mediated tissue destruction.

## Discussion

Here we describe a novel method to generate therapeutically relevant numbers of antigen-specific human Tregs. These TCR-redirected Tregs can be selectively enriched *in vitro* by reactivation with APCs and peptide to reach clinically viable cell numbers (i.e., in the range of 10^9^ cells) [12]. Lentiviral infected Tregs expand with similar kinetics to mock-infected Tregs and maintain the phenotypic and functional characteristics of nTregs *in vitro*. Specifically, antigen-specific Tregs suppressed Teff cells recognizing a common antigen (tyrosinase), in addition to bystander Teff cell responses. Importantly, the suppressive phenotype of human Tregs expressing the TyrTCR was recapitulated *in vivo* using murine Tregs and antigen-specific Teff cells.

Recent studies suggest that Tregs may exert direct tissue protective effects, in addition to their ability to limit Teff cell responses. Mechanistically, Tregs are thought to accomplish this through enzymatic pathways that reduce oxidative stress and produce anti-inflammatory byproducts [45], [46], [47], [48]. Here we show that Tregs can be directed to recognize MHC class I-restricted antigens allowing direct tissue recognition by transfer of a high-avidity TCR. This capability allows Treg activity to be directed to specific tissue microenvironments and target tissues deficient in APCs expressing MHC class II molecules. While not specifically addressed here, the use of TCR gene transfer to Tregs should facilitate studies addressing the optimal affinity of receptors as well as the lineage stability of Tregs in humanized animal model systems [49].

This technology opens up avenues for additional transgenes to be transferred in addition to the TCR as therapeutic gene cassettes. This approach could extend the function of engineered Tregs beyond that of even native Tregs. For example, the desired TCRs may be expressed along with a combination of immunoregulatory cytokines (IL-10 or TGF-β), chemokine receptors or tissue-homing receptors, or even tissue regenerative growth factors if necessary.

The use of viral gene transfer in Treg therapy settings confers the ability to address several important safety concerns. First, while a high purity of FOXP3^+^ Tregs was detected following our expansion procedure, the concern remains that an autoreactive TCR could potentially be expressed in a contaminating Teff cell or a Treg cell that has lost regulatory function [50]. Our approach could reinforce the regulatory program by the expression of FOXP3, or other regulatory genes in our expression cassette. Second, our system allows for the inclusion of inducible suicide genes or depletion markers should situations involving leukemia or pan-immunosuppression result.

One potential concern with this approach is the possibility for mismatched pairing of the introduced α and β TCR chains with the endogenous TCR subunits, resulting in reduced surface expression and biological activity. Mismatched pairing of receptors could potentially also generate harmful reactivities when expressed in effector T cell subsets. Cohen and colleagues previously addressed this issue by adding a single cysteine on each receptor chain to promote the formation of an inter-chain disulfide bond [51]. These cysteine-modified receptors retain their function and lead to preferential pairing, thus interfering with heterologous chain pairing between introduced and endogenous receptors [51]. Strategies such as codon optimization of TCR α and β chains have also been used to optimize *de novo* TCR expression over the endogenous receptors (reviewed [52]). The tyrosinase TCR used in our animal model experiments incorporated many of these modifications resulting in improved pairing and *in vivo* function.

In summary, novel gene therapy approaches, in concert with the previously outlined cellular therapeutics may offer new opportunities to generate antigen-specific tolerance by targeting the regulatory properties of Tregs to specific antigen targest. Prior studies employing adoptive cell therapy in the cancer setting imply that large numbers of transferred cells may be required in order to elicit a therapeutic effect [12], [53]. Here we demonstrated a strategy to efficiently generate therapeutic numbers of antigen-specific Tregs from low starting peripheral blood volumes. Applications of this strategy to engineer Tregs capable of recognizing autoantigens targeted in T1D are currently underway, with the goal of developing robust and targeted immunotherapies for the treatment of autoimmune diseases and transplantation.

## Materials and Methods

### Ethics Statement

Informed written consents for patient studies were obtained in accordance with the reviewed and approved policies and procedures at the University of California, San Francisco (UCSF), and ethics approval was granted by the UCSF Institutional Review Board (approval number H7023-22712-08). Animal studies were approved by the Animal Research Committee (ARC) of UCLA (approval number 2004-159) and the Institutional Animal Care and Use Committee (IACUC) of UCSF (approval number AN082188-01). Animals were bred and maintained according to the guidelines of the Department of Laboratory Animal Medicine (DLAM) at UCLA.

### Patient Population

Patient samples were collected from normal healthy adult subjects from the general population (N = 10; 5M/5F, median age 28.58 yr, range 20.71 to 34.71).

### Sample processing

Fresh peripheral blood was collected in sodium heparinized vacutainer tubes (BD; Franklin Lakes, NJ) and processed immediately for isolation of lymphocyte subpopulations. Whole blood was pre-enriched with the RosetteSep CD4^+^ negative selection cocktail and processed according to manufacturer recommendations (StemCell Technologies, Vancouver, BC, Canada). As an additional purification step, CD4^+^ T cells were incubated with CD45RO-biotinylated antibody (Leinco Technologies, St. Louis, MO; 0.1 µg/ml per 1×10∧6 cells). CD45RO-biotin labeled CD4^+^ T cells were then bound with BioMag streptavidin microbeads (50 ml/10^8^ cells) followed by a 5 minute incubation and subsequent depletion on the EasySep magnet (StemCell). Untouched CD4^+^CD45RA^+^ T cells were then resuspended at 1×10^8^ cells per ml and stained with antibodies in PBS containing 10% FBS for FACS isolation.

### Isolation of Tregs and Tconv cells by FACS

Tregs and Tconv cells were isolated on a BD FACSAria II high speed cell sorter following the CD4^+^CD45RA^+^ pre-enrichment (BD Biosciences, San Jose, CA) with the following antibodies: CD4-PerCP (clone SK3), CD127-PE (hIL-7R-M21), and CD25-APC (2A3) as previously described [24], with the addition of CD45RA-PE-Cy7 (HI100) to select the naive Treg subset. CD4^+^CD45RA^+^CD127^lo/-^CD25^+^ regulatory T cells (Tregs) and CD4^+^CD45RA^+^CD127^+^CD25^−^ conventional T cells (Tconv) were sorted using aseptic technique in a cGMP-level clean room facility and the sorted populations were collected into 2 ml of RPMI 1640 (Mediatech, Manassas, VA) complete media containing 10% heat-inactivated FCS. Populations were analyzed for purity post-sort and determined to be mean±SD, 98.5%±1.07; median 98.7%, range 95.8 to 99.7 for Tconv cells and 97.7±1.87; range 93.8–99.6% for Tregs.

### Lentiviral vectors for expression of TCRs

The generation of the tyrosinase TCR (TyrTCR) construct was previously described [34], [54]. Expression of the tyrosinase-reactive TCR α and β-chain genes in Treg and Tconv cells was achieved by cloning the TCR construct into the pSICO lentiviral vector [35]. This receptor is derived from a CD4^+^ T cell but functions independently of the co-receptor [34]. Equal molar expression of TCR α and TCR β-chain genes was made possible by the inclusion of a T2A expression element between the TCR α and β chain genes [35]. A 1857 bp EcoRI-XhoI TyrTCR fragment was generated by PCR from the original vector and ligated with a 1303 bp XhoI-NotI internal ribosome entry site (IRES) and eGFP reporter gene into the pSICO backbone. The pSICO construct was driven off the murine leukemia virus-derived MND promoter. All constructs were validated by restriction enzyme digest and sequence analysis.

### *In vitro* expansion procedure

FACS isolated cells were plated at 2.5×10^5^ Tregs or Tconv cells per well in a 24-well plate (Costar; Cambridge, MA) and activated with anti-CD3/anti-CD28 coated microbeads (Invitrogen; Carlsbad, CA) at a 1∶1 bead to cell. At 48 hr, cultures were split into mock transduced and lentiviral TCR transduced populations. Cultures were grown in the presence of IL-2 following day 2 and continuing throughout the 14-day expansion period (300 IU/ml, Proleukin; Chiron Therapeutics, Emeryville, CA). Cells were resuspended and fresh media and IL-2 (300 IU/ml) added at days 5, 7, 9, and 12 assuming consumption of IL-2. On day 9, cells were restimulated with fresh anti-CD3/anti-CD28 coated beads for mock transduced cells at a 1∶1 ratio or with artificial APCs (aAPC), a K562 cells line expressing HLA-A*0201, CD80, CD83 (A kind gift of Lee M. Nadler, Boston, MA) and soluble tyrosinase peptide (0.1 µg/ml, [Asp^370^]-Tyrosinase_(368–376)_; AnaSpec, Inc., San Jose, CA). Cultured cells were harvested at day 14 for analysis by FACS and functional studies.

### Lentiviral production and transduction of T cells

Lentivirus was produced as previously described [54]. Activated T cells were transduced 48 hr following polyclonal activation with anti-CD3 and anti-CD28 coated microbeads (Dynal, Invitrogen). 2.5×10^5^ cells per well were transduced in one ml total volume of fresh culture media in a 24-well plate containing lentivirus (20 TU/cell) and protamine sulfate (8 µg/ml; Sigma-Aldrich, St. Louis, MO). Cells were spinnoculated by centrifugation at 1000×*g* for 30 min at 32°C. Transgene expression was assessed no earlier than 72 hrs post-transduction.

### Phenotypic analysis of expanded human Treg populations

Freshly expanded cells were evaluated for continued expression of CD4 (SK3), eGFP, TCR Vβ12 (clone VER2.32.1) or HLA-A*0201 tyrosinase_(YMDGTMSQV)_ tetramer (Beckman Coulter, Fullerton CA). Anti-human FOXP3 (clone 206D) was purchased from BioLegend (San Diego, CA) and intracellular staining performed with the FOXP3 staining kit according to manufacturer's instructions and modified as previously described [25]. Flow cytometric data was collected on a FACSCalibur cytometer (BD Biosciences) and analyzed with FlowJo software (version 7.2.2, TreeStar, Ashland, OR). Epigenetic analysis of the *FOXP3*-TSDR was conducted by a methylation-sensitive real time PCR method developed by Epiontis Corporation (Berlin, Germany).

### *In vitro* stimulation and suppression assays

For the purposes of this study, suppression was assessed based on the capacity of *in vitro* expanded Tregs to suppress the proliferation of syngeneic Tconv cell populations. APCs were provided by either autologous CD3 depleted (RosetteSep CD3 depletion cocktail, StemCell) HLA-A2^+^ (irradiated 3300 rads) PBMC for polyclonal stimulation conditions or HLA-A2^+^/CD80^+^/CD83^+^ K562-cell derived artificial APCs (irradiated 10,000 rads) for antigen-specific suppression assays. For polyclonal suppression assays, cells were stimulated with 2 µg/ml soluble anti-CD3 (Hit3a) and 1 µg/ml soluble anti-CD28 (28.2, BD PharMingen). Soluble tyrosinase peptide_(368–376)_ was added at 0.1 µg/ml for measuring antigen-specific suppression assays, with melan-A peptide_(27–35)_ (MART-1) used as an HLA-A*0201-restricted negative control peptide (Anaspec). Cultures were incubated for 4 days at 37°C in a 5% CO_2_ incubator. Proliferation was determined by the incorporation of ^3^H-thymidine by pulsing cultures with 1 µCi of ^3^H-thymidine for the final 12–16 h of culture. Plates were harvested on a Packard FilterMate harvester and read on a Packard TopCount Scintillation & Luminescence Counter (Perkin Elmer; Waltham, MA). Percent suppression was calculated as previously described [55].

### Isolation and expansion of murine Tregs

CD4^+^CD25^+^CD62L^high^ Tregs were isolated from lymph nodes and spleens of 8–10 week old female C57BL/6 mice. Cells were pre-enriched with the mouse CD4^+^ T cell enrichment kit according to manufacturer's instructions (StemCell). Following enrichment, cells were stained with anti-mouse CD4-PE-Cy7, CD25-PE-Cy5, CD62L-FITC, and CD8-APC-Cy7 (as a dump fluorescence channel). Cells were sorted on a MoFlo Cell Sorter (Beckman Coulter) with two consecutive rounds of sorting to reach high purity prior to *in vitro* expansion (Median 99.4%, range 99.1–100). Cells were expanded *in vitro* by activation with Dynabead mouse T cell activator anti-CD3 and anti-CD28 coated microbeads (3∶1 bead to Treg ratio, Invitrogen) with exogenous IL-2 (2000 IU/ml, Novartis) as previously described [23].

### Retroviral transduction of murine T cells and adoptive cell transfer (ACT)

High-titer retrovirus stocks were prepared by transient co-transfection of Phoenix-Eco cells as previously described [56]. Retroviral constructs were based on a MSCV vector backbone containing a hybrid TyrTCR with complete murine constant region substitutions, and addition of cysteine residues on each chain as described [51]. Leucine zipper motif sequences were added to the 3′ terminus of each chain to improve pairing and 2A sequences inserted between the TCR α, β chain, and marker transgenes [35]. For effector T cell generation, cells from spleen and lymph nodes of 8 weeks-old HLA-A2/K^b^ transgenic mice were harvested and RBC-depleted with Red Blood Cell Lysing Buffer (Hybri-Max, Sigma, St. Louis, MO). Cells were then incubated in anti-CD3 and anti-CD28-coated plates (BD Biosciences) for activation of T cells. At 48 hours post-activation, cells underwent spinoculation with retrovirus supernatants (10 multiplicity of infection) in retronectin (Takara-bio, Shiga, Japan)-coated plates at 1000×g, 120 minutes, 32°C, in a Beckman CS-6R centrifuge. The transduction procedure was repeated again following 24 hours and incubated overnight. Cells were then washed and re-suspended in culture media for analysis or in PBS for injection. Eight-week old HLA-A2/K^b^ transgenic mice were injected subcutaneously (s.c.) with EL4-A2/K^b^ cells expressing tyrosinase protein were used for adoptive cell transfer when the tumors reached 5 mm in diameter. Mice were conditioned with myeloablation (900 cGy total-body irradiation), followed by rescue with intravenous injection of 10^6^ total bone marrow cells. On the next day (day 0), mice were randomized into control groups with Treg cells and T effector cells transduced with control mock vector and treatment group with Treg cells transduced with Tyr-TCR/TK/GFP vector for intravenous injection. Dendritic cells (DC) were differentiated from bone marrow progenitor cells obtained from HLA-A2/K^b^ mice by *in vitro* culture in murine granulocyte macrophage colony-stimulating factor (GM-CSF, 50 ng/ml) and murine IL-4 (50 ng/ml; R&D Systems, Minneapolis, MN) as described [57]. DCs were harvested as loosely adherent cells, washed twice in PBS (Mediatech) and pulsed with tyrosinase_(368–376)_ peptide at a concentration of 10 µM in serum-free media for 90 minutes at room temperature. Each mouse received 10^5^ pulsed DCs given s.c. on day 0. On day 2, all mice received T effector cells transduced with Tyr-TCR/firefly (f)Luciferase vector intravenously. Recombinant human IL-2 (250,000 I.U.) was injected intraperitoneally on days 0, 1 and 2. HLA-A2/Kb transgenic mice were originally obtained as a kind gift from Dr. Linda Sherman (Scripts, La Jolla, CA).

### Luciferase-based in vivo Bioluminescence Imaging (BLI)

BLI was performed with a Xenogen IVIS 200 Imaging System (Xenogen/Caliper Life Sciences, Hopkinton, MA). Mice were anesthetized with 2% isoflurane and injected with D-luciferin (Xenogen; 1,500 µg per animal) for imaging of fLuc-transduced T cells. Imaging was performed 5 minutes later and analyzed with Living Image 2.50 software (Xenogen). The time of acquisition varied from 3 to 5 minutes. The mean number of photons per square centimeter per second per steradian in the region of interest (ROI) was determined on tumor-implanted sites.

### Cell culture

*In vitro* T cell expansion and suppression assays were conducted in RPMI 1640 media (Mediatech) supplemented with 5 mM HEPES, 2 mM L-glutamine, penicillin/streptomycin (50 µg/ml each) (Invitrogen, Carlsbad, CA), 50 µM 2-mercaptoethanol (Sigma), 5 mM non-essential amino acids, 5 mM sodium pyruvate (Mediatech), and 10% FCS (Invitrogen). For suppression assays, cultures were maintained in 200 µl volume in U-bottom 96-well plates (Costar, Cambridge, MA) incubated at 37°C and 5% CO_2_.

*Cytokine Analysis*. Supernatant was collected from TCR transduced Jurkat T cells and analyzed for IL-2 production by standard sandwich ELISA according to manufacturer recommendations (BD Biosciences).

### Statistics and methods of analysis

Multiple group comparisons were conducted by ANOVA with Bonferroni post tests. Data analyses utilized GraphPad Prism 5.00 software (GraphPad, San Diego, CA, USA) and values at *P*<0.05 were deemed significant. Cytokine concentrations were determined using SOFTmax® PRO software (Molecular Devices, Sunnyvale, CA) with four-parameter data analysis.

## Supporting Information

Figure S1 Representative FACS plots showing sorted human CD4+CD25+CD45RA+ Treg and CD4+CD25-CD45RA+ Tconv T cells. Human peripheral blood was collected in sodium heparin vacutainer tubes. CD4+ T cells were enriched by incubating whole blood (80 ml) with the CD4 negative selection cocktail (50 µl/ml) followed by ficoll density gradient centrifugation. Following CD4 enrichment, cells were incubated with anti-human CD45RO-biotinylated antibody (0.1 µg/1×10̂6 cells) followed by magnetic depletion with streptavidin-coated microbeads. The resulting negatively selected CD4+CD45RA+ T cell population was stained for CD4, CD25, CD127, and CD45RA and sorted on a FACS Aria II cell sorter. Shown are pre- and post-sort expression of (A–C) CD4 (x-axis) and CD45RA (y-axis) and (D–F) CD127 (x-axis) and CD25 (y-axis) on Treg and Tconv cell populations, as indicated. (G) CD4+CD45RA+CD127-/loCD25+ Treg (N = 8) or CD4+CD127+CD25- Tconv cells (N = 4) were analyzed for FOXP3 expression by flow cytometric analysis following 14 d of in vitro expansion and by real-time PCR method for percent demethylated at the FOXP3-TSDR. No significant difference in percent positive by FACS (mean +/− SD, 92.3+/−9.6) and percent demethylated at the FOXP3-TSDR (90.9+/−15.3) was observed for Tregs, whereas Teff cells exhibited significantly higher levels of FOXP3 protein (43.6+/−25.3) versus (1.0+/−1.3) percent demethylated-FOXP3-TSDR (P<0.05).
(1.70 MB TIF)

Figure S2 Lentiviral transduction does not alter the capacity of Tregs to expand or suppress T responder cell proliferation. Fresh human CD4+CD127-CD25+ T cells were isolated by FACS and expanded over a 14 day culture period with two rounds of anti-CD3 & anti-CD28 coated beads and exogenous IL-2 (300 IU/ml). 48 h following activation, either mock treated or lentiviral transduced cells expressing GFP (9 TU/cell) were expanded to day 14. At day 14, pSICO-R.eGFP [54] lentiviral transduced cells were FACS sorted into transgene negative (−), (low), and (high) expressing cells (A, left column). (B) The suppressive capacity of each of these sorted cells was then tested at various ratios of Tregs to T responder cells (1∶1, 1/2∶1, 1/4∶1, 1/8∶1, and 1/16∶1; where 1 = 5×10∧4 T cells) as indicated. For suppression assays, experiments were conducted in triplicate using freshly isolated autologous CD3+ T cell depleted APCs (1×10∧5 cells/well, irradiated 3300 rads) in the presence of soluble anti-CD3 (2 µg/ml) and anti-CD28 (1 µg/ml). (C) The capacity of lentiviral transduced cells (green triangles) to expand in vitro is not reduced compared to mock treated Tregs (black squares).
(0.19 MB TIF)

Figure S3 Expression of tyrosinase TCR constructs by in vitro expanded human Treg and Tconv cells. Initial expression of surface TCR, GFP, and FOXP3 following a nine day in vitro expansion period for mock or TyrTCR transduced Treg and Tconv cells. Cells were stimulated with anti-CD3 and anti-CD28 microbeads for 48 hours prior to lentiviral spinoculation. Plots indicate expression of eGFP (y-axis) and TCR Vβ12 or HLA-A2 tyrosinase(368–376) tetramer staining (x-axis) with (A, upper plots) representing mock transduced cells and (B, lower plots) showing TyrTCR transduced populations.
(0.48 MB TIF)

Figure S4 TyrTCR Tregs produce IL-10 in response to peptide activation and suppress Teff cell production of IL-2 and IFN-γ. TyrTCR or Mock Tregs were incubated with the tumor infiltrating Teff cell clone (TIL1235) at indicated ratios in the presence of peptide pulsed HLA-A2.1 T2 APCs. Culture supernatants following 24 hours were harvested and analyzed for production of IL-2 (blue), IFN-γ (red), and IL-10 (green). Bars indicate cytokine detected in co-cultures with mock Tregs (filled bars) and TyrTCR Tregs (open bars).
(0.80 MB TIF)
