# Supplementary figures and images for: Human Antigen-Specific Regulatory T Cells Generated by T Cell Receptor Gene Transfer
Source: PLoS One. 2010 Jul 22;5(7):e11726. doi: 10.1371/journal.pone.0011726 (PMC2908680; doi:10.1371/journal.pone.0011726)

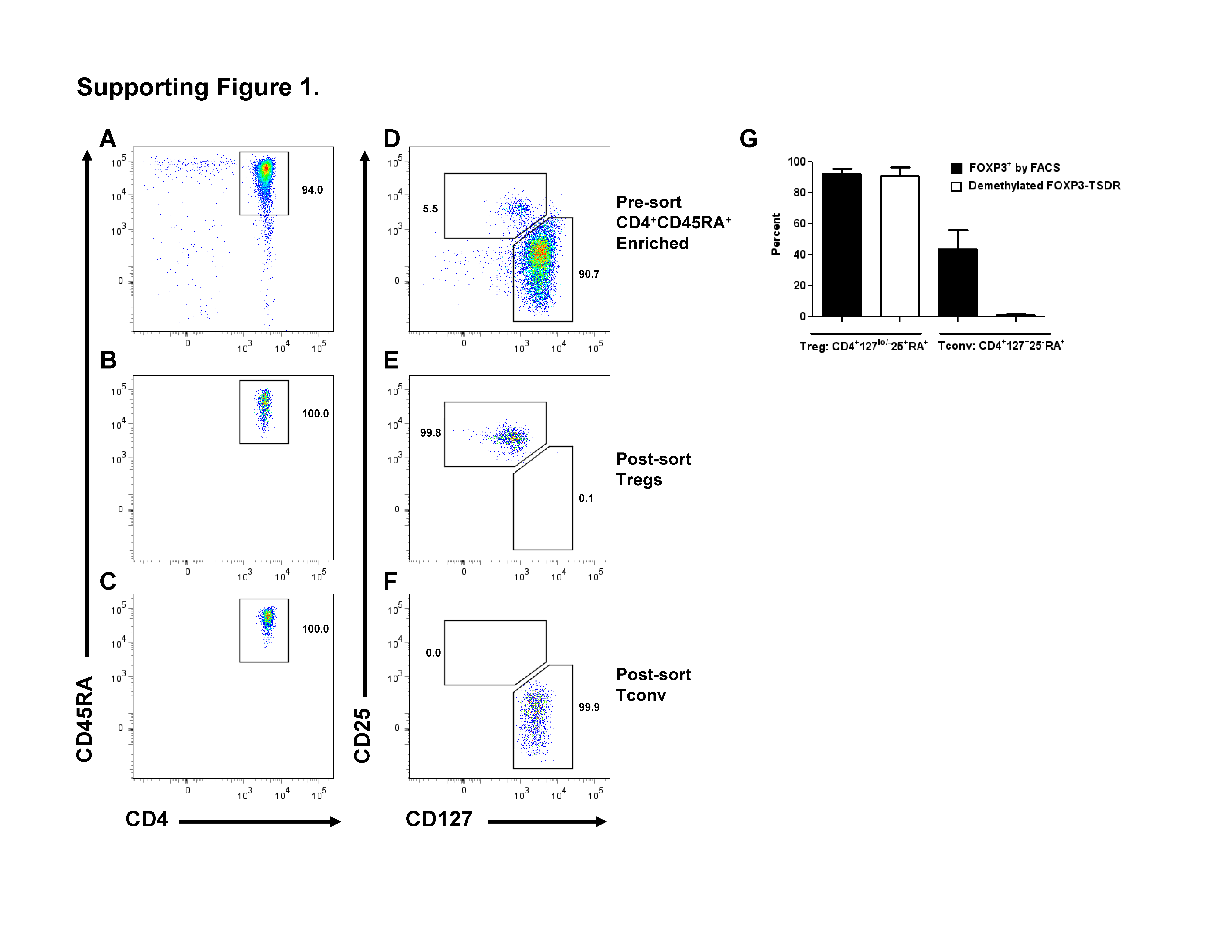

Supplement: Figure S1 — Representative FACS plots showing sorted human CD4+CD25+CD45RA+ Treg and CD4+CD25-CD45RA+ Tconv T cells. Human peripheral blood was collected in sodium heparin vacutainer tubes. CD4+ T cells were enriched by incubating whole blood (80 ml) with the CD4 negative selection cocktail (50 µl/ml) followed by ficoll density gradient centrifugation. Following CD4 enrichment, cells were incubated with anti-human CD45RO-biotinylated antibody (0.1 µg/1×10̂6 cells) followed by magnetic depletion with streptavidin-coated microbeads. The resulting negatively selected CD4+CD45RA+ T cell population was stained for CD4, CD25, CD127, and CD45RA and sorted on a FACS Aria II cell sorter. Shown are pre- and post-sort expression of (A–C) CD4 (x-axis) and CD45RA (y-axis) and (D–F) CD127 (x-axis) and CD25 (y-axis) on Treg and Tconv cell populations, as indicated. (G) CD4+CD45RA+CD127-/loCD25+ Treg (N = 8) or CD4+CD127+CD25- Tconv cells (N = 4) were analyzed for FOXP3 expression by flow cytometric analysis following 14 d of in vitro expansion and by real-time PCR method for percent demethylated at the FOXP3-TSDR. No significant difference in percent positive by FACS (mean +/− SD, 92.3+/−9.6) and percent demethylated at the FOXP3-TSDR (90.9+/−15.3) was observed for Tregs, whereas Teff cells exhibited significantly higher levels of FOXP3 protein (43.6+/−25.3) versus (1.0+/−1.3) percent demethylated-FOXP3-TSDR (P<0.05). (1.70 MB TIF) [file pone.0011726.s001.tif]

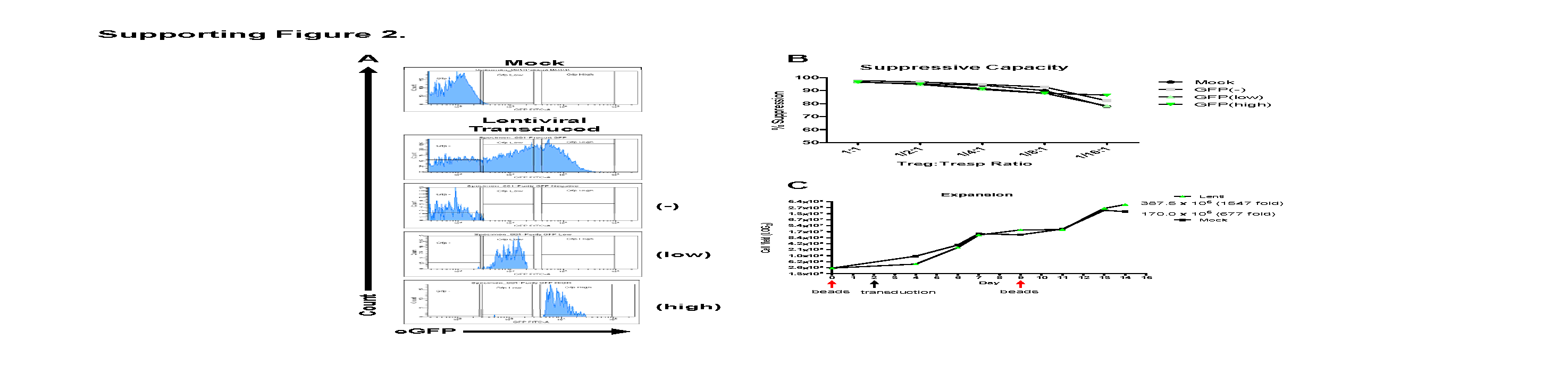

Supplement: Figure S2 — Lentiviral transduction does not alter the capacity of Tregs to expand or suppress T responder cell proliferation. Fresh human CD4+CD127-CD25+ T cells were isolated by FACS and expanded over a 14 day culture period with two rounds of anti-CD3 & anti-CD28 coated beads and exogenous IL-2 (300 IU/ml). 48 h following activation, either mock treated or lentiviral transduced cells expressing GFP (9 TU/cell) were expanded to day 14. At day 14, pSICO-R.eGFP [54] lentiviral transduced cells were FACS sorted into transgene negative (−), (low), and (high) expressing cells (A, left column). (B) The suppressive capacity of each of these sorted cells was then tested at various ratios of Tregs to T responder cells (1∶1, 1/2∶1, 1/4∶1, 1/8∶1, and 1/16∶1; where 1 = 5×10∧4 T cells) as indicated. For suppression assays, experiments were conducted in triplicate using freshly isolated autologous CD3+ T cell depleted APCs (1×10∧5 cells/well, irradiated 3300 rads) in the presence of soluble anti-CD3 (2 µg/ml) and anti-CD28 (1 µg/ml). (C) The capacity of lentiviral transduced cells (green triangles) to expand in vitro is not reduced compared to mock treated Tregs (black squares). (0.19 MB TIF) [file pone.0011726.s002.tif]

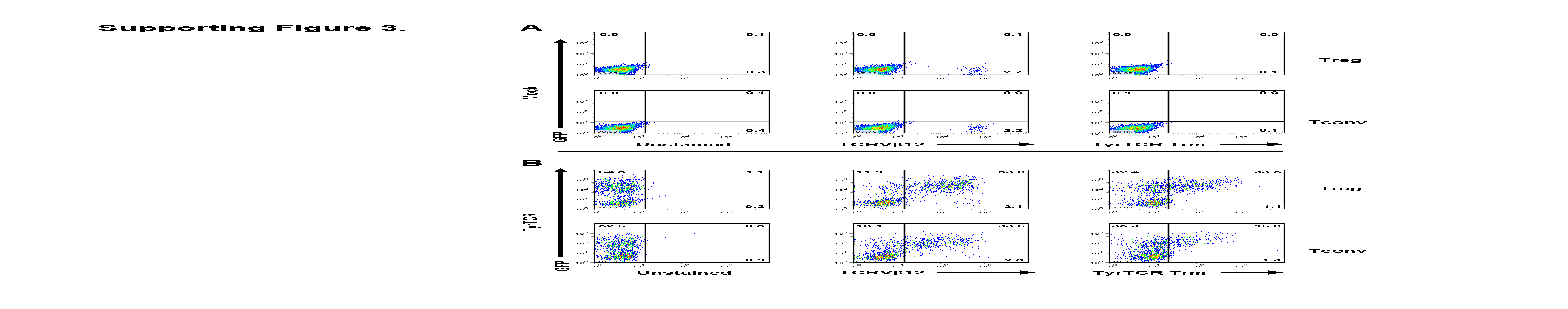

Supplement: Figure S3 — Expression of tyrosinase TCR constructs by in vitro expanded human Treg and Tconv cells. Initial expression of surface TCR, GFP, and FOXP3 following a nine day in vitro expansion period for mock or TyrTCR transduced Treg and Tconv cells. Cells were stimulated with anti-CD3 and anti-CD28 microbeads for 48 hours prior to lentiviral spinoculation. Plots indicate expression of eGFP (y-axis) and TCR Vβ12 or HLA-A2 tyrosinase(368–376) tetramer staining (x-axis) with (A, upper plots) representing mock transduced cells and (B, lower plots) showing TyrTCR transduced populations. (0.48 MB TIF) [file pone.0011726.s003.tif]

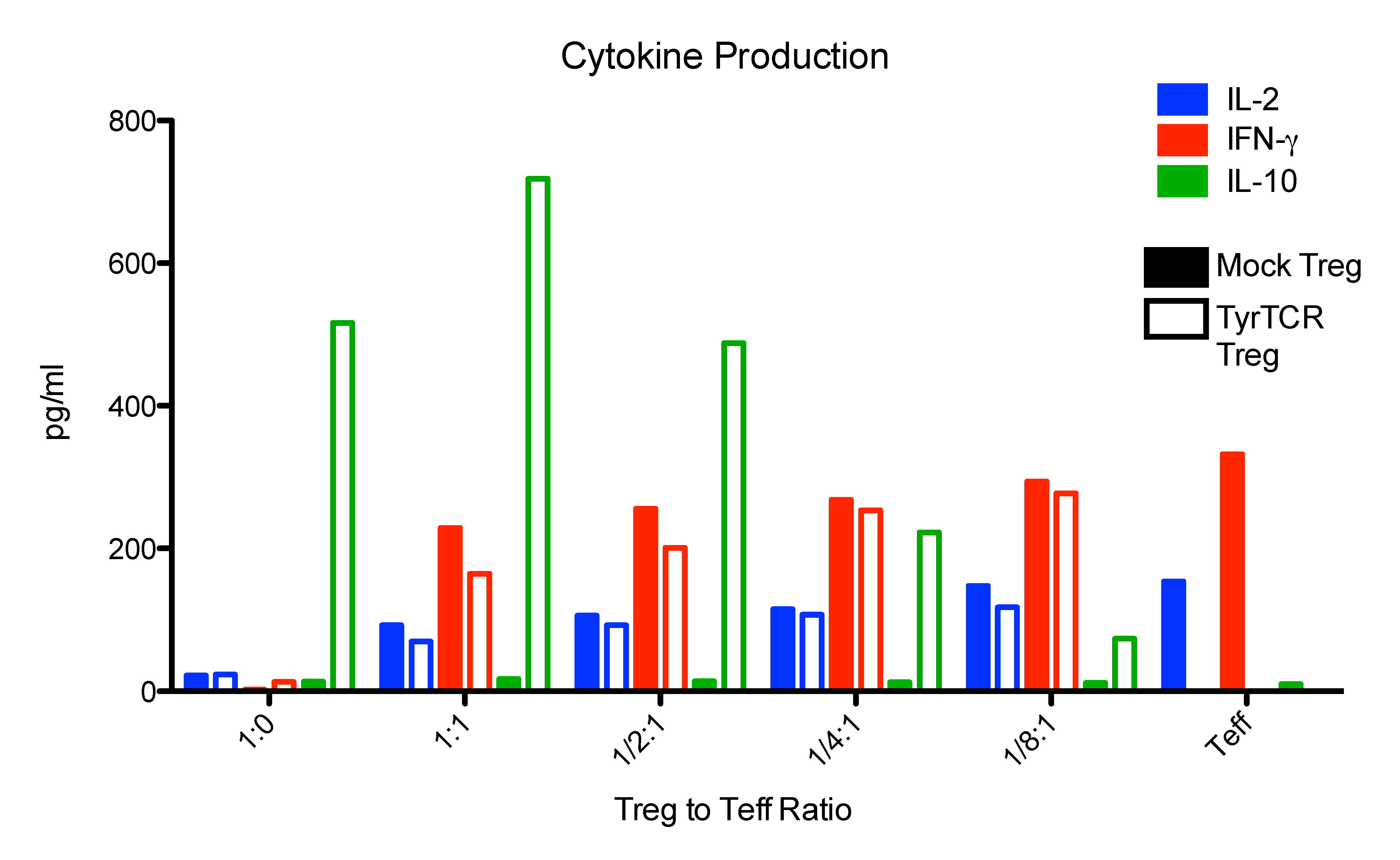

Supplement: Figure S4 — TyrTCR Tregs produce IL-10 in response to peptide activation and suppress Teff cell production of IL-2 and IFN-γ. TyrTCR or Mock Tregs were incubated with the tumor infiltrating Teff cell clone (TIL1235) at indicated ratios in the presence of peptide pulsed HLA-A2.1 T2 APCs. Culture supernatants following 24 hours were harvested and analyzed for production of IL-2 (blue), IFN-γ (red), and IL-10 (green). Bars indicate cytokine detected in co-cultures with mock Tregs (filled bars) and TyrTCR Tregs (open bars). (0.80 MB TIF) [file pone.0011726.s004.tif]
